# Supplementary material for: Non-proliferative neurogenesis in human periodontal ligament stem cells
Source: Sci Rep. 2019 Dec 2;9:18038. doi: 10.1038/s41598-019-54745-3 (PMC6888846; doi:10.1038/s41598-019-54745-3)
Supplement: Supplementary file 1 — Supplementary information [file 41598_2019_54745_MOESM1_ESM.pdf]

Supplementary information

Title: Non-proliferative neurogenesis in human periodontal ligament stem cells.

Authors: Carlos Bueno[1*], Marta Martínez-Morga[2] and Salvador Martínez[1].

[1] Instituto de Neurociencias de Alicante (UMH-CSIC), San Juan, Alicante, 03550, Spain.

[2] Department of Human Anatomy and Institute of Biomedical Research (IMIB), University of Murcia, Faculty of Medicine, Murcia, 30800, Spain.

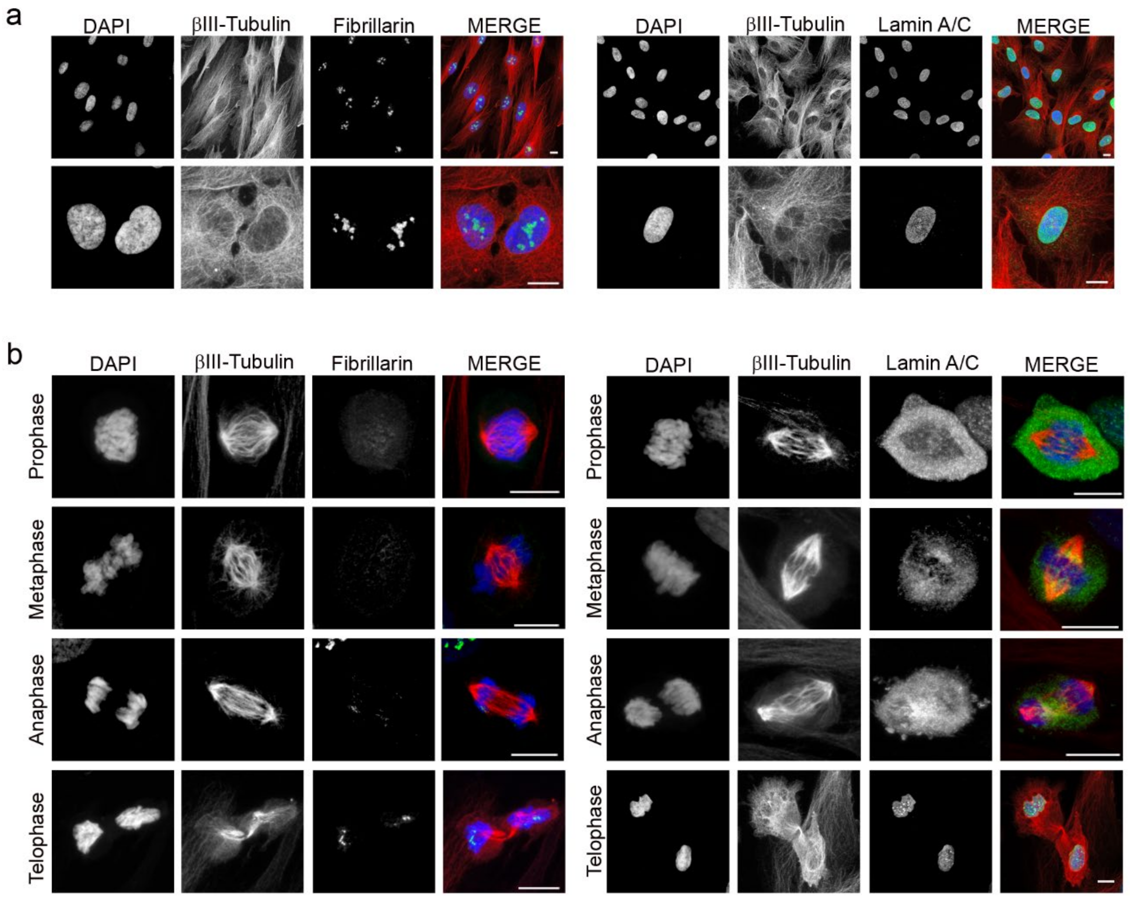

Figure S1. Dynamic localization of fibrillarin and lamin A/C proteins during the cell cycle of proliferative hPDLSCs. (**a**) During interphase, the nuclei of hPDLSCs contained two or more nucleoli and the inside surface of the nuclear envelope is lined with the nuclear lamina. (**b**) The nuclear lamina and nucleolus are reversibly disassembled during mitosis. Scale bar: 10 μm.

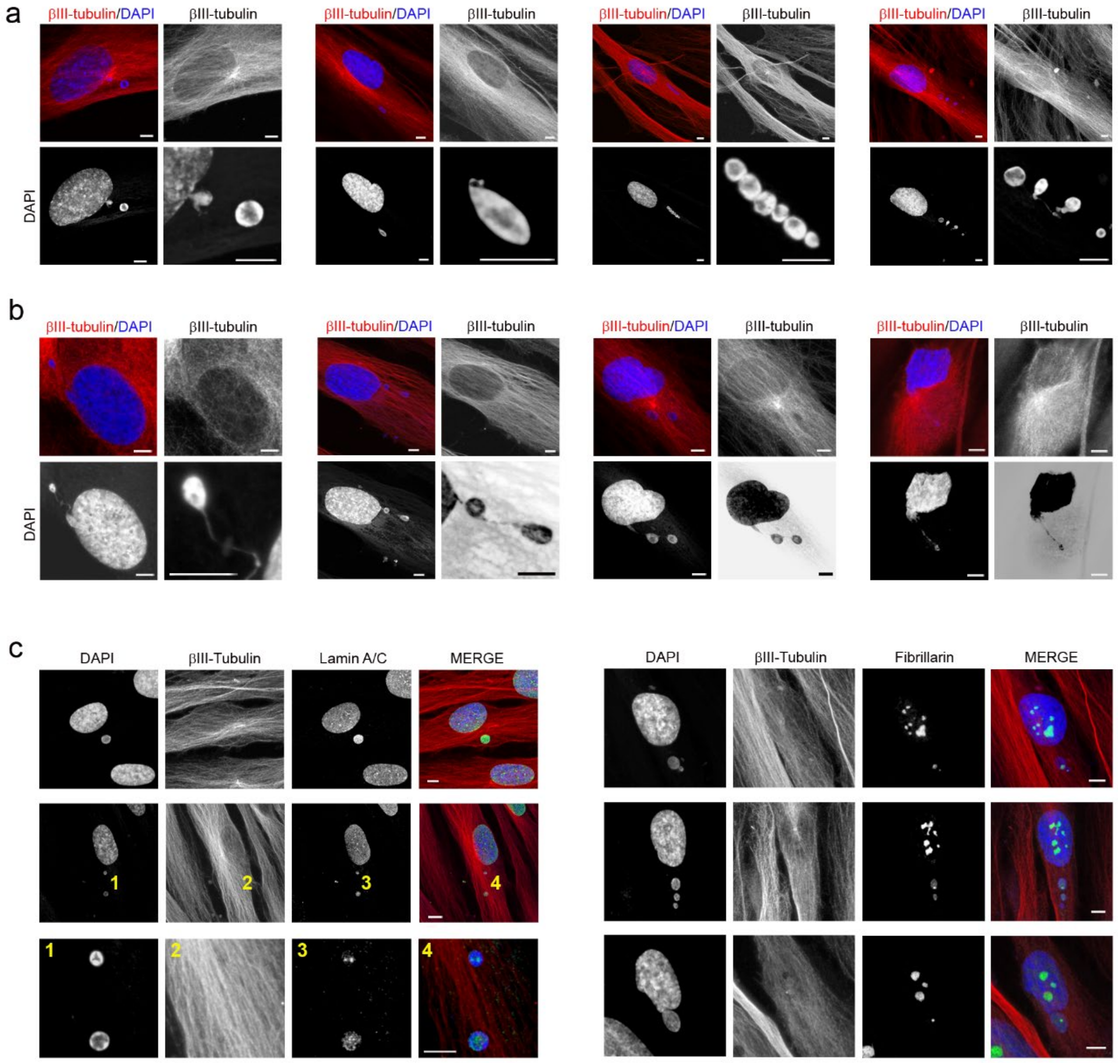

Figure S2. Cytoplasmic DNA containing structures. (**a**) Cytoplasmic DNA containing structures displayed a spherical or ovoid shape and it seems that some of them are connected to the main body of the nucleus by thin strands of nuclear material (**b**). (**c**) Fibrillarin and lamin A/C proteins were detected in small DNA containing structures (numbers locate the areas showed in higher power). Scale bar: 5 µm.

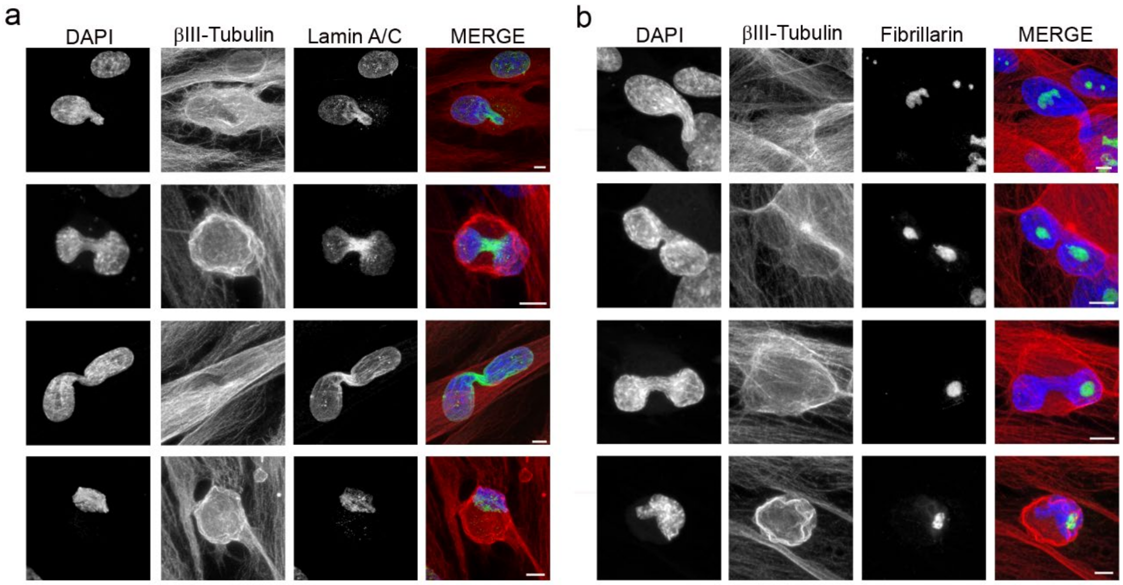

Figure S3. Dynamic localization of fibrillarin and lamin A/C proteins during in vitro neurogenesis from hPDLSCs. The nuclear lamina (**a**) and nucleolus (**b**) are not disassembled during in vitro neurogenesis from hPDLSCs. Futhermore, β-III tubulin is not present in the mitotic spindle. Scale bar: 5 μm.

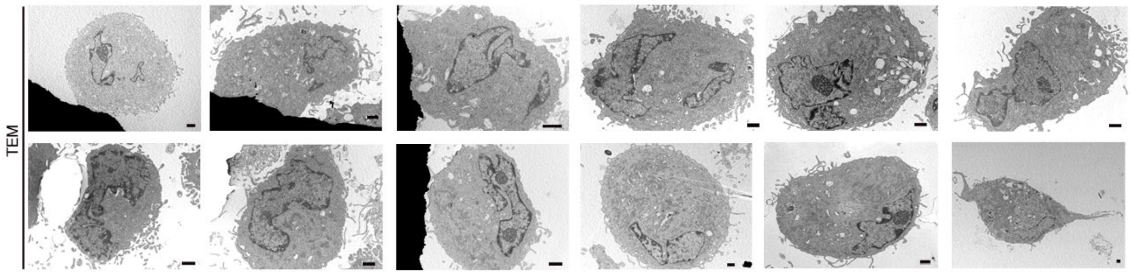

Figure S4. hPDLSCs with lobed nuclei do not represent apoptotic cells. Transmission electron micrographs show that hPDLSCs with abnormal nuclei do not represent apoptotic cells due to the absence of features of cells undergoing apoptosis. Scale bar: 1 μm. TEM, transmission electron microscopy.

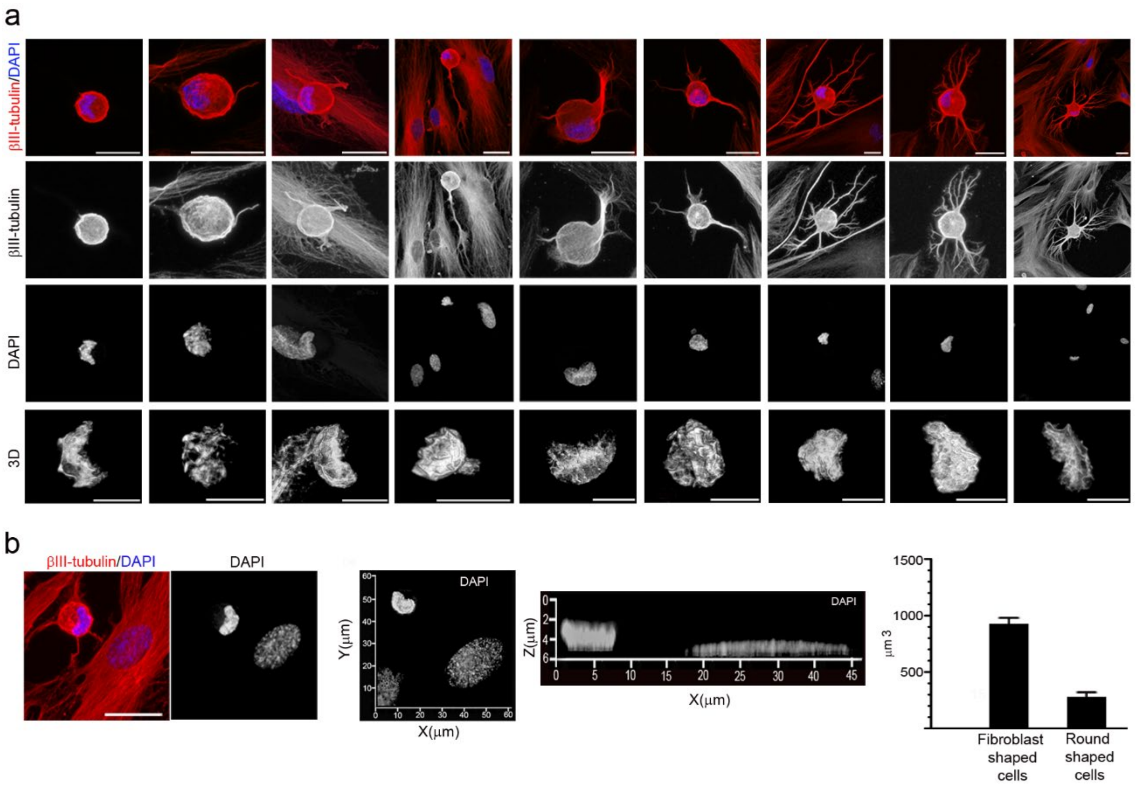

Figure S5. Nuclear shape in hPDLSCs-derived neural-like cells during neuronal polarization. (**a**) No lobed nuclei are observed when hPDLSCs-derived neural-like cells gradually acquired cellular polarity and more mature, neural-like morphology. (**b**) The nuclear volume shrinks as the cells become rounded during neurogenesis. Data represent mean ± S.E. of ten independent experiments. The scale bar in β-III tubulin and DAPI images are 50 μm and 10 μm for confocal 3D images of nuclei.
